# Feasible Self-Calibration of Larger Field-of-View (FOV) Camera Sensors for the Advanced Driver-Assistance System (ADAS)

**DOI:** 10.3390/s19153369

**Published:** 2019-07-31

**Authors:** Vijay Kakani, Hakil Kim, Mahendar Kumbham, Donghun Park, Cheng-Bin Jin, Van Huan Nguyen

**Affiliations:** 1 Information and Communication Engineering, Inha University, 100 Inharo, Nam-gu Incheon 22212, Korea; 2 Valeo Vision Systems, Dunmore Road, Tuam, Co. Galway H54, Ireland; 3 Faculty of Information Technology, Ton Duc Thang University, Ho Chi Minh City 758307, Vietnam

**Keywords:** advanced driver-assistance system (ADAS), larger field-of-view (FOV), self-calibration, radial distortions, parameter sharing, model-specific empirical γ-residual rectification factor

## Abstract

This paper proposes a self-calibration method that can be applied for multiple larger field-of-view (FOV) camera models on an advanced driver-assistance system (ADAS). Firstly, the proposed method performs a series of pre-processing steps such as edge detection, length thresholding, and edge grouping for the segregation of robust line candidates from the pool of initial distortion line segments. A novel straightness cost constraint with a cross-entropy loss was imposed on the selected line candidates, thereby exploiting that novel loss to optimize the lens-distortion parameters using the Levenberg–Marquardt (LM) optimization approach. The best-fit distortion parameters are used for the undistortion of an image frame, thereby employing various high-end vision-based tasks on the distortion-rectified frame. In this study, an investigation was carried out on experimental approaches such as parameter sharing between multiple camera systems and model-specific empirical γ-residual rectification factor. The quantitative comparisons were carried out between the proposed method and traditional OpenCV method as well as contemporary state-of-the-art self-calibration techniques on KITTI dataset with synthetically generated distortion ranges. Famous image consistency metrics such as peak signal-to-noise ratio (PSNR), structural similarity index (SSIM), and position error in salient points estimation were employed for the performance evaluations. Finally, for a better performance validation of the proposed system on a real-time ADAS platform, a pragmatic approach of qualitative analysis has been conducted through streamlining high-end vision-based tasks such as object detection, localization, and mapping, and auto-parking on undistorted frames.

## 1. Introduction

Recently, use of vision-based sensors such as cameras and LiDARs on autonomous platforms have become ubiquitous as they provide the perception of the 3D world around us. Advanced Driver-Assistance System (ADAS) is one such platform which heavily relies on the performance of these sensor units for its stable endurance [1]. Some sensors such as LiDAR units can be effective in depth perception yet highly expensive compared to their affordable counterparts such as monocular and stereo cameras. In reality, it is more efficient and affordable to use multiple camera systems instead of multiple LiDAR units on the vehicle. Also, recently the usage of wide-angle and fisheye camera systems has become almost mandatory on ADAS platforms for better coverage area. This approach substantially increased the performance of ADAS tasks in terms of understanding the 3D world around the vehicle as well as path planning [2]. Additionally, with better visual cues and larger field of view (FOV)—these wide-angle/fisheye camera systems are much more feasible to operate alongside narrow FOV cameras on tasks such as object detection, tracking, simultaneous localization and mapping (SLAM) etc. [3]. Such feasibility demands a certain amount of tolerance towards numerous practical issues such as lens distortions, specifically radial lens distortions. Generally, radial distortions vary according to the lens models and their configurations such as fisheye, wide-angle, and a super-wide-angle lens. These variations in lens modeling and specificity make it inflexible for modeling and estimating a common distortion factor for a collective set of lens models [4]. In practice, it is most unlikely even for the two cameras of the same lens model to possess similar distortion parameters, which makes the concept of distortion parameter into a camera-specific distinctive feature [5].

### 1.1. Previous Studies

There is a significant amount of literature on estimating and rectifying the lens distortions in the context of larger FOV lens models. Larger FOV lens models such as fisheye and wide-angle require precise arbitrary calibration target object with known metrics to retrieve the alterations among pixels in lens projections corresponding to the known target object metrics [6]. Usually, this approach can be considered to be manual calibration which is highly prone to procedure-induced errors such as shape irregularities of the calibration targets (imperceptible bending of checkerboard pattern which influences projection of the squared-box edges on to the camera lens). Therefore, precautionary measures should be taken for the calibration scenario to be precise and well-organized to achieve accurate lens calibration [7]. In the context of multiple camera systems used on ADAS platforms, the calibration of an individual sensor unit demands a well-ordered approach and an extra amount of tuning time. With the increase in the demand for exploitation of larger FOV cameras on ADAS platforms [8], industries and companies tend to explore more feasible approaches to calibrate such lens, eluding human intervention and extensive manual labor [9]. Specifically, automobiles are often exposed to various temperatures and climatic conditions which can heavily influence the dynamics of intrinsic parameters of the cameras attached to them [10,11]. Under such circumstances, lens-distortion parameters estimated using pattern-based pre-calibration method cannot adapt to those conditions rising a need for re-calibration with specific calibration object. To solve these hurdles, there exist many approaches in the literature attempting to totally eliminate the usage of any physical object in the calibration process [12,13].These methods are regarded as “autocalibration” or “self-calibration” approaches [14,15,16] which are able to estimate the distortion parameters without the need for any pre-defined calibration object. These self-calibration techniques are further classified into various categories depending upon their approaches such as absolute conic-based lens-distortion rectification [17], absolute quadric, and circular arc–based autocalibration [18,19] etc.

Among them, the widely popular approach is the plumb-line method of lens calibration which relies on various scene geometrical attributes such as straight lines, vanishing points, edges, and man-made objects such as buildings, road lanes, etc. This pioneering study by [20] suggested formulating the radial distortions using polynomial lens-distortion model by rectifying curved lines in the image domain which are actually straight in the real world. Later, a few other studies proposed numerous models yet one other significant model which is as famous as Brown’s polynomial model is the one-parameter rational model proposed by [21,22]. Technically, self-calibration algorithms employ certain optimization techniques to estimate distortion parameters concurrently while fitting curved distorted lines to its real straight lines at a pixel-level [23]. A few studies, such as Barreto et al. [24] and Zhang et al. [25] presented automatic techniques to calibrate the camera systems. However, limitations such as “scene must contain at least single chessboard pattern”, “need for perpendicularly placed structured patterns for the estimation of low rank texture matrix”, etc. limits the real-time feasibility. Various other methods were proposed to estimate parameters from the visual attributes of a scene such as vanishing points and straight lines. The plumb-line techniques and vanishing point approach demands the scene to have at least a few parallel lines with any two of them being collinear [26].

Very recently, the plumb-line techniques employed by various approaches demonstrated the algebraic exploitation of the division models [27] as well as scene lines to estimate the parameters with intense iterative optimizations [28]. The fundamental idea in most of these successful self-calibration techniques is to involve a loss function which minimizes the distortion effect with a constraint on straight lines. Theoretically, this demands heavy optimization algorithms to track the constraints on straight lines (making them appear straight in image domain as they are in the real world) and simultaneously estimating the distortion parameters corresponding to the specific lens. In many ways, those algorithms work on specific lens models. However, these algorithms fail to rectify the pixel irregularities in the presence of severe distortions and often produce unstable outputs. Some methods might actually need a severe pre-testing to determine the tuning parameters required for the intermediate procedures which are indeed the building blocks for the main algorithm [29,30].

### 1.2. Purpose of Study

**Figure 1:**
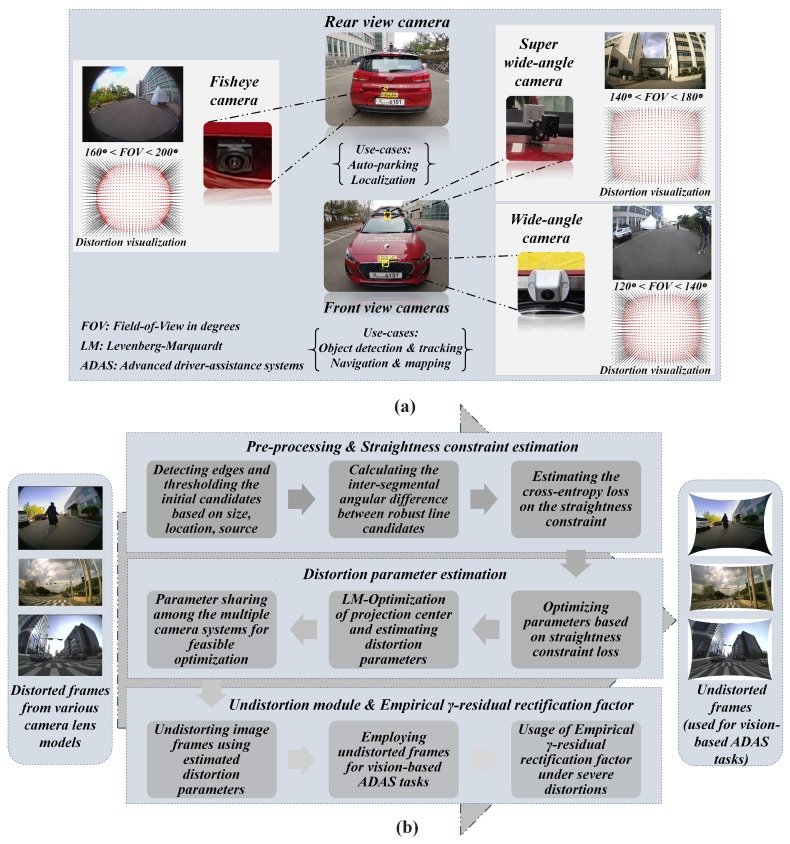
Proposed Pipeline on ADAS workbench (**a**) ADAS Platform: Camera sensors setup and image acquisition. (**b**) Proposed method with block schematics.

The main objectives of this study are: (1) to formulate a novel straightness constraint loss with effective pre-processing approaches that can yield better distortion rectification on various larger FOV camera lens under diverse conditions, (2) to validate the self-calibration system on ADAS workbench for the betterment of higher vision-based tasks such as object detection, localization, mapping, auto-parking etc. and finally (3) to examine various practical aspects such as parameter sharing, model-specific empirical γ-residual rectification factors for an optional usage in worst-case scenarios such as severe distortions and huge image sizes. In this study, various lens models such as fisheye (160${}^{\circ }$ < FOV < 200${}^{\circ }$), wide-angle (120${}^{\circ }$ < FOV < 140${}^{\circ }$) and super-wide-angle (140${}^{\circ }$ < FOV < 180${}^{\circ }$) were experimented and tested under diverse real-time scenarios. The ADAS system used in this study is depicted in Figure 1 illustrating the camera sensors, image acquisition setup, and proposed system flow. All the camera models and image acquisitions with distortion visualizations are shown in the top half of the Figure 1. In the bottom half of the Figure 1, the block schematic of the proposed method is shown which contains three blocks internally.

In the first block, the scene geometry was exploited to retrieve the edge line segments which constitute the majority of the straight line segments in the real scene. The pre-processing steps such as thresholding based on size, location and source (originating from the same edge structure) were employed to retrieve the initial line segments. These line candidates are then used to calculate the inter-segmental angular difference as a straightness constraint and a cross-entropy loss is estimated. In the second block, the straightness constraint loss is used alongside LM-optimization to estimate the distortion parameters by minimizing the error. By leveraging the shared ADAS workbench, estimated parameters are then shared across the other camera models thereby assisting them in effortless optimization. The final undistortion block rectifies the distorted frame using estimated parameters and this block is used in parallel with vision-based ADAS tasks running on those undistorted frames. Additionally, model-specific empirical γ-residual rectification factors can be employed for undistortion along with normal mode of undistortion in the presence of severe distortions. These empirical factors were estimated with various examinations on multiple camera models targeting for better stretch-free image quality.

**Figure 2:**
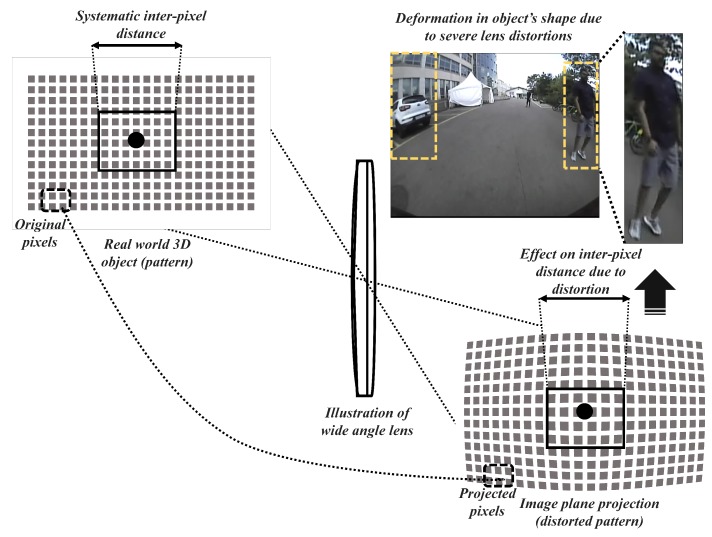
Structural anomaly induced into a scene due to heavy lens distortion caused by wide-angle cameras with field-of-view 120${}^{\circ }$ < FOV < 140${}^{\circ }$.

Furthermore, the outcomes of this study were concentrated towards the real-time ADAS vision-based scenarios to explore practical hurdles in the context of employing object detectors, navigators on larger FOV sensors. Because vision-based tasks such as object detection, localization, and tracking, etc. need to be performed on the camera retrieved image samples which must be distortion-free. The image projections from a larger FOV camera lens often suffer from severe radial distortions which can alter the perception of 3D real-world objects in the 2D image domain. The variations in inter-pixel distance is directly proportional to the lens distortion and lens FOV [31,32]. In practice, this affects the shape of objects observed in the scene thereby imposing enormous uncertainty for tasks such as object detection and tracking, localization, and mapping. This analogy can be observed in Figure 2, where the pixels corresponding to a human in the 3D real-world scene is heavily distorted and projected on to the image plane.

Most ADAS systems circumvent this problem by avoiding the distortion correction process in the context of fisheye or wide-angle cameras and training deep-learning models using raw distorted image samples for the object detection and tracking tasks etc. [33,34]. These models trained from scratch using those distorted samples can perform well on distorted test data which might affect their performance on evaluation datasets such as KITTI or any other open datasets which were acquired using various other camera models [35,36]. Additionally, tasks such as depth estimation, SLAM, and 2D-3D metric estimation rely on distortion-free samples for the approximation of pixel-to-real-world correspondences [37]. Practically, these issues demand a solid need for a self-calibration approach which can rectify the distortions of the larger FOV lens images and improve the feasibility in using them for ADAS-based vision tasks. Therefore, this study validates the proposed method with an extensive amount of practical testing on vision tasks such as object detection, localization, auto-parking, and point-cloud consistency. The main contributions of the study are:

- The novel straightness constraint was formulated on the pre-processed candidates with a cross-entropy loss function used to define the problem of error minimization.
- The robustness of the proposed system was extensively validated in real-time testing on various vision-based ADAS tasks under numerous scenarios.
- An experimental attempt of using parameter sharing approach and model-specific empirical γ-residual rectification factors has been investigated and the limitations of the proposed system were analyzed for future betterment.

The rest of this paper is organized as follows. Section 2 briefly elaborates the lens modeling concepts and explains the polynomial lens-distortion model used in this study. Section 3 describes the detail implementation of the proposed self-calibration design. Section 4 illustrates the experimental data collection and synthetic data generation scenarios. Section 5 describes the performance analysis and evaluation metrics used for the validations. Section 6 presents the results and discussions corresponding to various experiments conducted on real and synthetic data. Finally, this paper is concluded in Section 7 with a summary.

## 2. Lens-Distortion Modeling

The projection of a real-world 3D scene by a standard camera is often mathematically modeled using a simple pinhole model [38]. The 3D object with coordinates (A,B,C) in the real world is projected through the lens on to the image plane in correspondence to a 3D Euclidean coordinate system. In the image domain, the 3D object coordinates are projected on to the 2D domain in the form of point coordinates (a,b) as shown in Equation (1) below.

(1) $$\begin{array}{c} a=\lambda_{a}u+sv+a_{0},\\ b=\lambda_{b}v+b_{0}, \end{array}$$

where skew factor is (*s*), focal length is (λ), image coordinates u=A/C, v=B/C and principal point is $\left(a_{0},b_{0}\right)$.

**Figure 3:**
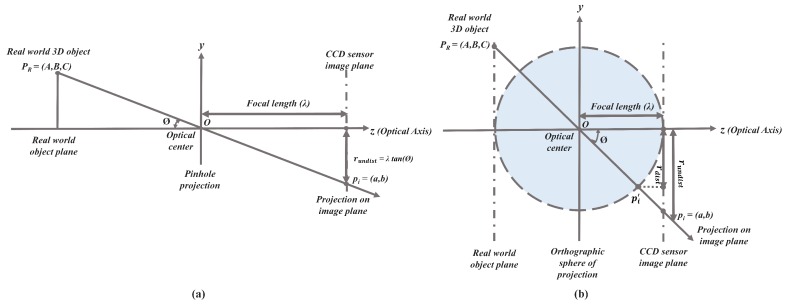
Lens Projection Models: (**a**) Standard Camera Pinhole Projection Model. (**b**) Larger FOV Lens Orthogonal Projection Model.

The pinhole standard projection model can better estimate the projection transformation [39] in the absence of lens distortions. However, there are several distortion aspects that might be inculcated at the time of manufacturing and handling lens, making the projection rays deviate from the optical axis corresponding to a non-linear lens-distortion function *D*. In the case of larger FOV cameras, the projection ray originating from the 3D object is heavily distorted on the image plane leaving the distorted pixels far from the principal point causing severe barrel distortion effect [40].The effect of barrel distortion is introduced by the inherited orthogonal sphere of projection phenomenon which projects the object $P_{R}=\left(A,B,C\right)$ on to the image plane as $p_{i}^{\prime }$ instead of $p_{i}$. This exact phenomenon is observed in a much larger FOV lens, especially in the fisheye lens and super-wide-angle lens. The projection phenomenon in the case of a standard pinhole model and larger FOV orthogonal sphere of projection is shown in Figure 3 below for better comparison.

The non-linear lens-distortion function distorts the pixel points pushing them away from the center causing the barrel effect with respect to the center of distortion $\left(a_{0},b_{0}\right)$. The below Equation (2) shows the distorted coordinates in correspondence to the center of distortion.

(2) $$\begin{array}{c} a=\lambda_{a}u_{dist}+sv_{dist}+a_{0},\\ b=\lambda_{b}v_{dist}+b_{0}, \end{array}$$

where $\left(u_{dist},v_{dist}\right)=D\left(u,v\right)$.

### Polynomial Lens-Distortion Model

These severe radial distortions must be modeled using an appropriate radial lens-distortion model that can estimate the distortions with a better approximation of the non-linear function D(r). Previous works on modeling radial lens distortions proposed various models that can approximate the distortions imposed by wide-angle and fisheye lens, eventually leaving the scope of distortion estimation to choose from numerous lens distortion models. One among the most commonly used model for estimating lens distortion is a polynomial radial distortion model which uses a polynomial distortion function D(r) as shown in Equation (3) to transform between distorted and undistorted pixels in correspondence to the radii (which is the Euclidean distance from the distortion center to a distorted point).

(3) $$D\left(r\right)=r\cdot \left(1+D_{1}\cdot r+D_{2}\cdot r^{2}+\cdots D_{N}\cdot r^{N}\right)=r\cdot \left(1+\sum_{n=1}^{N}D_{n}\cdot r^{n}\right),$$

where $r\left(radius\right)=\sqrt{{\left(a-a_{0}\right)}^{2}+{\left(b-b_{0}\right)}^{2}}$ and $D_{1},D_{2},\cdots D_{N}$ are distortion coefficients.

The above equation is a more generalized formulation of polynomial lens-distortion model. Specifically, the even and odd polynomial variants can be used to model the equation for distortion estimation.

Even polynomial lens-distortion model:

(4) $$D\left(r\right)=r+D_{1}\cdot r^{2}+D_{2}\cdot r^{4}+\cdots =r\cdot \left(1+\sum_{n=1}^{N}D_{n}\cdot r^{2\cdot n-1}\right),$$

Odd polynomial lens-distortion model:

(5) $$D\left(r\right)=r+D_{1}\cdot r^{3}+D_{2}\cdot r^{5}+\cdots =r\cdot \left(1+\sum_{n=1}^{N}D_{n}\cdot r^{2\cdot n}\right).$$

In this study, the distortion estimation and optimization procedures were followed as per the odd polynomial lens-distortion model with up to two distortion coefficients $D_{1},D_{2}$ which maps rectified pixel coordinates to the distorted pixel coordinates as shown in Equation (6) below.

(6) $$r_{dist}=r_{undist}+D_{1}\cdot r_{undist}^{3}+D_{2}\cdot r_{undist}^{5}=r_{undist}\left(1+D_{1}\cdot r_{undist}^{2}+D_{2}\cdot r_{undist}^{4}\right).$$

## 3. Proposed Self-Calibration Design

**Figure 4:**
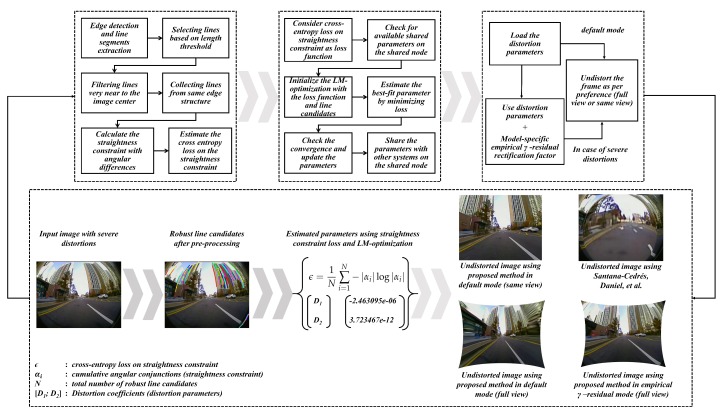
Proposed Self-calibration design.

The proposed self-calibration design consists of three blocks namely: pre-processing and estimation of straightness constraint loss, parameter estimation using LM-optimization and straightness constraint loss minimization, undistortion module using estimated distortion parameters. Apart from the major attributes of the proposed system, an empirical study has been made to analyze the worst-case scenarios in larger FOV lens models and a simple aspect such as parameter sharing and gamma-residual hyperparameter has been suggested for model-specific lens models. The detailed flow of the proposed system is illustrated in the Figure 4 below which also include block-wise outputs. The input heavy distorted 165${}^{\circ }$ FOV image was depicted on purpose in the Figure 4 to depict the worst-case scenario of distortion rectification. The undistortion outputs were estimated and to give a perspective of distortion severity, the same input has been undistorted using the state-of-the-art self-calibration method [28].

### 3.1. Pre-Processing and Straightness Constraint Loss

**Figure 5:**
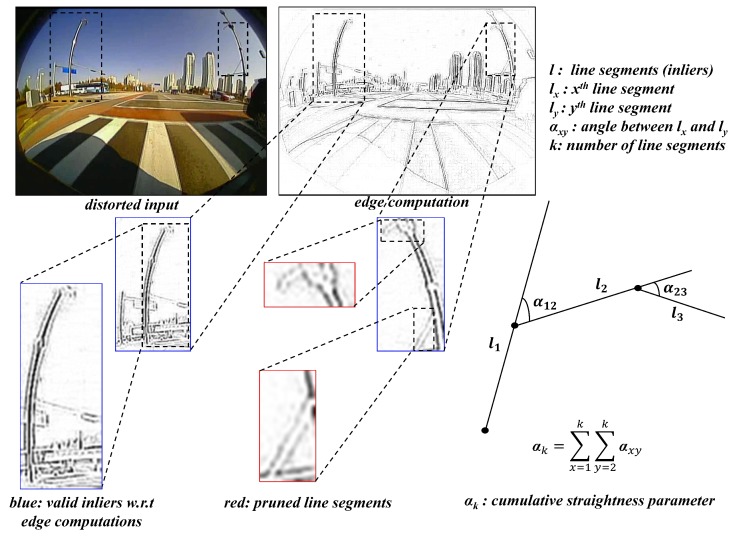
Pre-processing of line candidates and Estimation of Straightness constraint.

The initial pre-processing stage was designed to gather the robust line candidates and this stage starts with edge detection using the edge drawing library [41] and segregates the robust line candidates from the observed scene based on length threshold (size of the line segments), location (lines very close to the center). The pre-processing of line candidates is depicted in Figure 5, where the blue segments are the ones considered to be the robust candidates and the red are the ones pruned as they do not meet the thresholds and edge groups.

After the filtering process, estimating the angular conjunctions between these segments emerging from the same structural entity and mathematically minimizing the angularity can result in maximizing the straightness property between them. This straightness constraint approach was carried out to formulate a loss function minimizing the cumulative error between the candidates. As the angular differences in terms of straightness constraint fluctuate between certain absolute values, the cross-entropy loss function is formulated for error handling, which appears to be more appropriate. The straightness constraint has been estimated and a cross-entropy loss has been imposed as shown in Equations (7) and (8).

(7) $$\alpha_{k}=\sum_{k=1}^{N}∥a\tan2\left(l_{k}\right)-a\tan2\left(l_{k}+1\right)∥,$$

(8) $$\epsilon =\frac{1}{N}\sum_{i=1}^{N}-\left|\alpha_{i}\right|\log\left|\alpha_{i}\right|,$$

where α is cumulative angular conjunctions with *k* as index of the line segment; *N* as total number of robust line candidates; ϵ being the cross-entropy minimization on straightness constraint.

### 3.2. Error Minimization and Distortion Parameter Estimation

**Figure 6:**
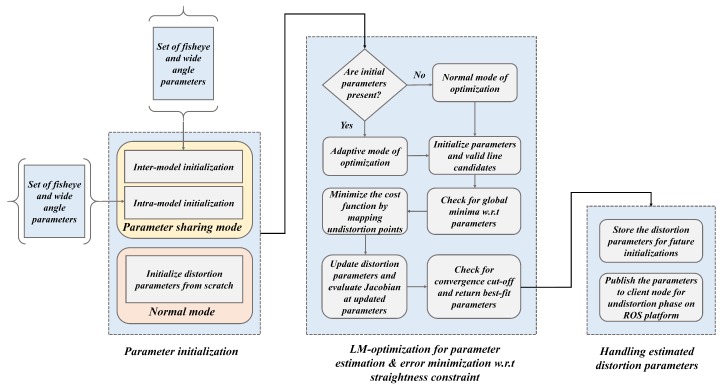
Schematic of distortion parameter estimation using LM-optimization in normal mode and parameter sharing mode.

The distortion parameter estimation in the proposed design was carried out by minimizing straightness constraint loss over LM-optimization. The loss estimated from the robust line candidates was considered to be the error function in the optimization step and concurrently, the distortion parameters were estimated. Leveraging the shared environment among multi-camera sensors enabled the parameter sharing for feasible optimization on multiple nodes on ADAS workbench. The whole procedure of distortion estimation and parameter optimization can be divided into three sub-modules as illustrated in the Figure 6.

The parameter estimation through LM-optimization starts off in two ways: one is the normal mode, in which the optimization is carried out normally with null value initialization and converging to the best-fit parameters. The second method is using parameter sharing approach of initializing the preliminary parameters in two intuitive ways: inter-model initialization and intra-model initialization. Specifically, the inter-model and intra-model mode of parameter initializations are selectively chosen depending upon the lens models. As the ADAS platform bears distinctive lens models such as wide-angle, fisheye, and super-wide-angle lens at the same time in operation, the use of parameter sharing for initialization can come in handy to reduce the enormous load on optimization. The proposed system performs the self-calibration of a certain camera and retrieves the parameters in the normal mode of optimization. Those parameters are further shared with the other camera models on-board for effortless optimization. During the optimization, the robust line candidates are used to minimize the loss function which in other words, the center of distortion is rectified by mapping undistortion points with respect to the straightness constraint. The estimated parameters are handled by sharing them on to a subscriber node, such that they can be stored for further optimization initializations as well as undistort the image frame such that vision-based tasks such as object detection, localization can be carried out on the distortion-rectified (undistorted) frames.

#### 3.2.1. Ablation Study Validating the Attributes in Distortion Parameter Estimation

**Figure 7:**
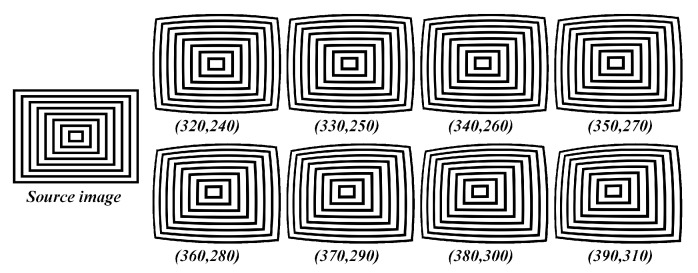
Synthetic data used in the ablation study validating the attributes of distortion parameter estimation.

This ablation study section was carried out in the process of evaluating the system under various loss functions and optimization schemes to validate the best possible approach both in terms of performance and processing time. The synthetic dataset shown in the Figure 7 was open-sourced by [27] with diverse distortion ranges and ground-truth principal points, therefore it has been used to perform the validation process. The metrics such as average error in principal point estimation (difference between original and predicted), average structural similarity index in undistorted image quality and parameter estimation times were considered on the synthetic dataset with several distortion variants and ground-truth principal points.

The below Table 1 gives a perspective of how different approaches achieved trade-offs between performance and processing time. In the below table, the following acronyms are used NR: Newton–Raphson, LM: Levenberg–Marquardt, M-S: Mean-squared, L-S: Least squares, C-E: Cross-entropy. The LM-optimization with cross-entropy loss was significant in maintaining the trade-off.

#### 3.2.2. Investigation of Parameter Sharing Option in Higher-Resolution Frame Processing

**Figure 8:**
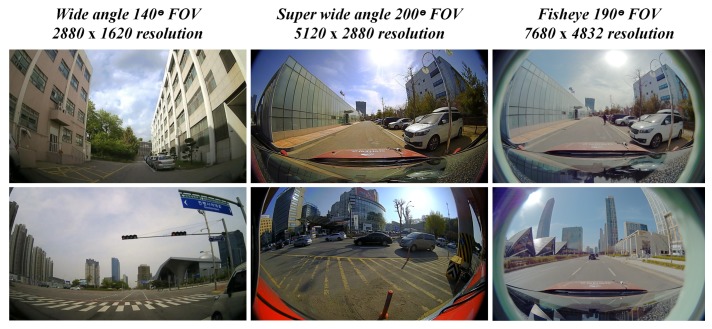
High-Resolution acquisitions of real-time data samples from larger FOV cameras.

The parameter sharing option during optimization can be invoked in the presence of high-resolution frames such as ultra HD, 4K and >4K. The investigation of parameter sharing option has been carried out on real-time data samples from larger FOV lens cameras at high resolutions. The Figure 8 below shows the data samples at various resolutions that are higher than usual. The FOVs of the cameras used for this investigation are 140${}^{\circ }$, 190${}^{\circ }$ and 200${}^{\circ }$ with all the three samples of different resolutions.

**Figure A1:**
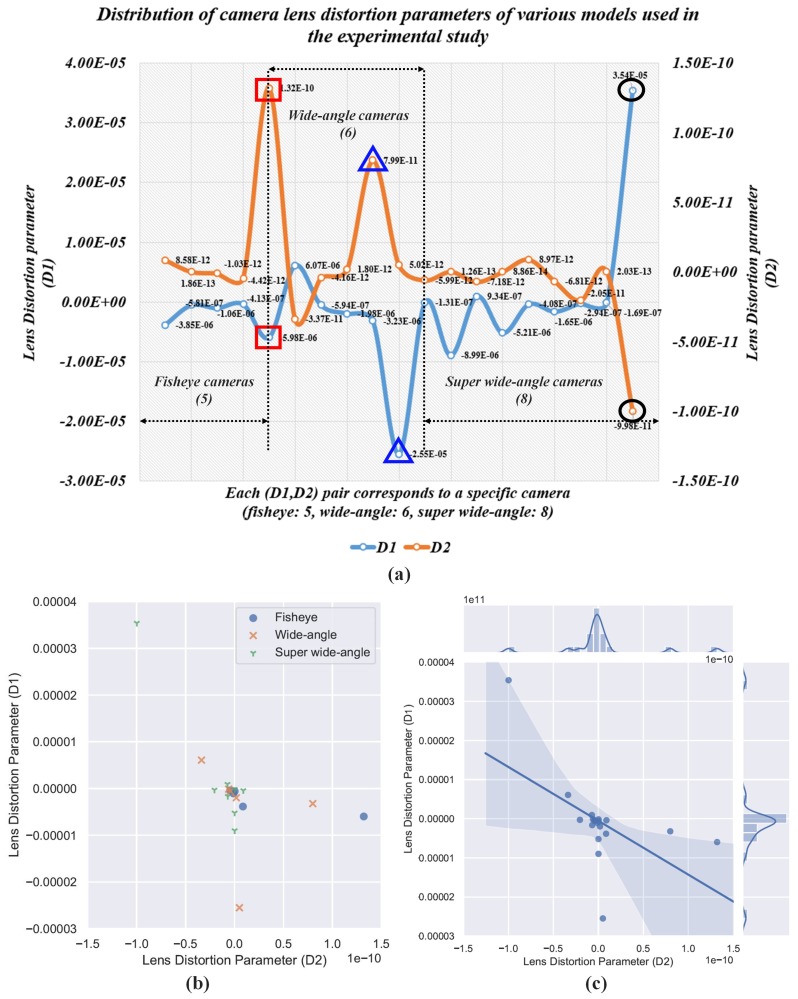
Distortion distributions: (**a**) $D_{1}$,$D_{2}$ parameters of various camera sensors. (**b**) $D_{1}$,$D_{2}$ distributions w.r.t camera models. (**c**) Regression plot to investigate the common region spread of $D_{1}$ and $D_{2}$.

The parameter sharing option was invoked and tested to record the estimation times. Table 2 presents the relative estimation times in each mode, parameter sharing option can come in handy while handling high-resolution image frames. Usually, the parameter sharing option can be added as a simple yet practical step during the main algorithm of estimating straightness constraint loss and distortion parameters as shown in Algorithm 1. Although the three types of lenses are classified into separate models, they fall under a single larger FOV lens-distortion pattern of heavy distortions along the edges and comparatively low at the center as shown in the Appendix A regarding the investigation of camera distortion distributions used in the study. As the polynomial lens-distortion model has a potential in handling distortions of such sorts, the real data distortions in this study were modeled and optimized by imposing the straightness constraint on them so as to rectify the distortions of all larger FOV lenses as a whole. The only practical difference between the three types of lenses in terms of calibration process is the handling of parameter sharing in case of larger size inputs and choice of γ-empirical hyper parameter in case of heavy distortions. Apart from those aspects, rest all the calibration process runs the same as described by the proposed pipeline for all the larger FOV camera models. Although it is unlikely even for the two cameras of the same lens model to possess similar parameters as shown in Figure A1, at least by sharing the parameters through inter-model and intra-model strategies: the execution time can be reduced by through forcing the optimizer to reach global minimum on the straightness constraint in shortest time possible.

**Algorithm 1:** Straightness constraint loss and Distortion parameter estimation.

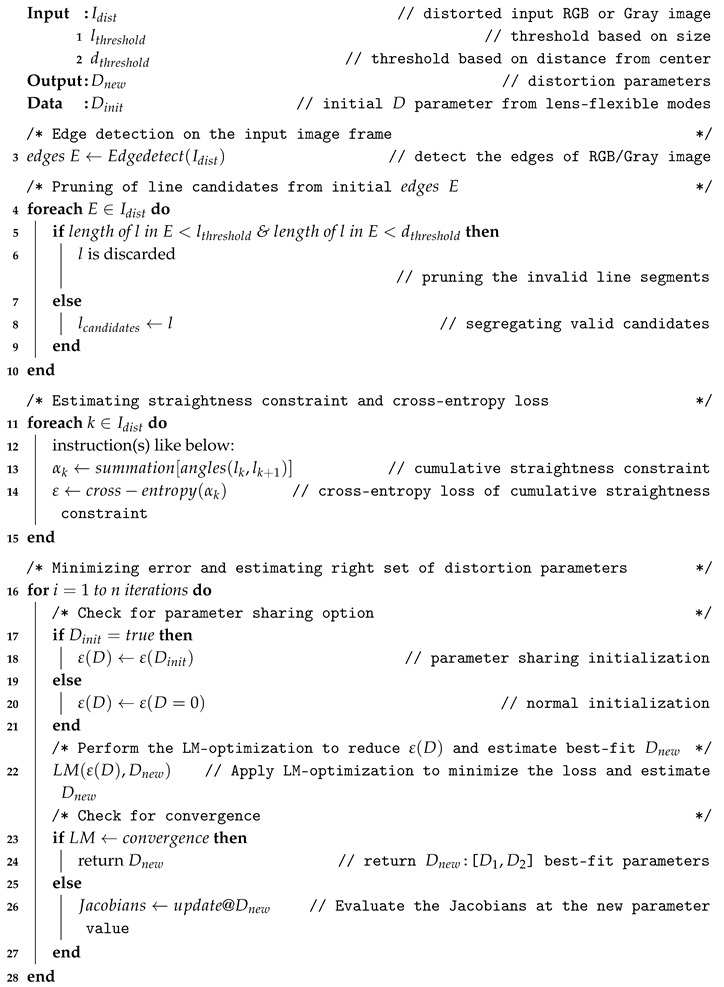



### 3.3. Undistortion Module

**Figure 9:**
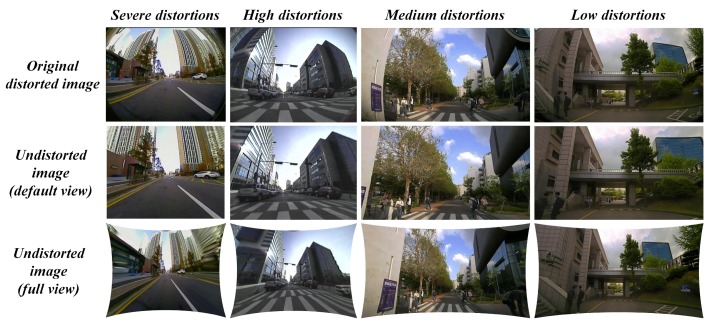
Distortion rectification of severe, high, medium, and low level of distortions using the proposed method.

The undistortion module in the proposed system acts as an initial process for other ADAS-based vision tasks running on the image frame. After the distortion parameters are estimated, the undistortion module uses those parameters to rectify the distorted image frames. The general mode of undistortion rectifies the distortions into two different views namely default view and full view. In default view: the output distortion-rectified frame appears to be same size as input and contains valid pixels without any stretching. In full view: the output distortion-rectified frame contains all the pixels so that the pixels along the edges appear stretched. The full view undistorted frame is often used in many ADAS applications to explore the blind spots around the vehicle using larger FOV cameras. The Figure 9 below illustrates the distortion-rectified outputs in hierarchical distortion levels such as severe, high, medium, and low. The proposed system with default undistortion mode works effectively with most of the distortion levels except in the “full view” of severe distortion level. The undistorted frame of the severely distorted image appears to have a stretching effect along the edges in the full view. These kinds of severe distortions were encountered during this study due to ultra-low-quality cameras lens exceeding 165${}^{\circ }$ of FOV with heavy distortions along the edges.

### 3.4. Model-Specific Empirical γ-Residual Rectification Factor

**Figure 10:**
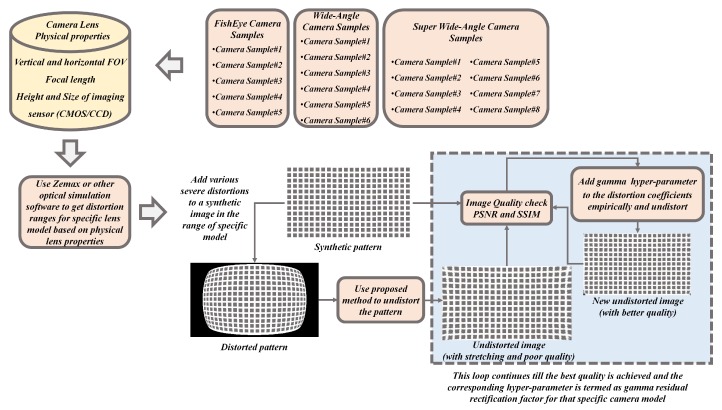
Empirical process of estimating model-specific γ-residual rectification factor.

The model-specific empirical γ-residual rectification factor is an experimental study attribute involved in this proposed system to overcome the severe distortions for particular set of camera models used in the context of this study. Most of the undistortion procedure is generally done using the default mode with parameters estimated by the proposed system. In some worst-case scenarios where the lens distortions are severe, then a model-specific empirical γ-residual factors can be used. This empirical approach is strictly optional and heavily dependents on the lens design. The process of deriving γ-residual factors is brute force in nature and can be achieved through extensive empirical trials on the lens models with known lens information such as FOV, size, and height of the imaging sensor etc. The procedure of empirically estimating model-specific γ-residual rectification factor is explained in Figure 10.

In this study, 3 sets of camera systems (5 fisheye lens, 6 wide-angle lens, 8 super-wide-angle lens) were extensively tested to empirically estimate the γ factors. The model approximate distortion ranges were defined using camera lens physical properties such as vertical and horizontal FOV, focal length, height and size of the imaging sensor in an optical software such as ZEMAX [42]. A normal synthetic image pattern was distorted using the range of distortions retrieved from optical software for the specific camera model. The distorted image is then undistorted using the proposed method and image quality metrics were calculated to check the undistortion quality. If the image quality is less than a certain threshold, the gamma hyperparameter is added to the distortion parameter and undistortion is performed in a loop simultaneously calculating the image quality metrics to yield the best possible output. This loop continues till the best quality is achieved and the corresponding hyperparameter is considered to be empirical γ-residual rectification factor for that specific camera model. The gamma factors of −0.2, −0.25 and −0.3 were assessed as pre-defined hyper parameters for wide-angle, super-wide-angle, and fisheye lens cameras, respectively.

**Figure 11:**
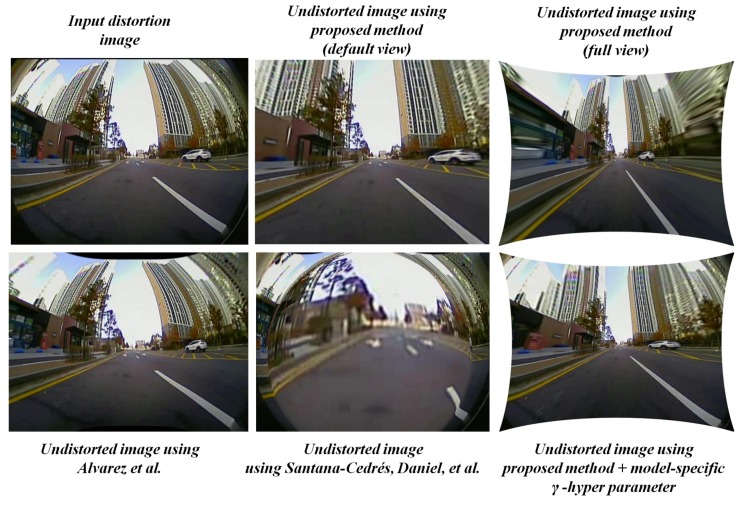
Severe distortion cases rectified using several approaches [28,29], proposed method with and without empirical γ-hyper parameter.

In real time, most of the undistortion was carried out by default mode of undistortion using parameters obtained from the proposed system. Only in a few cases as shown in Figure 11 were the empirical factors employed to solve the issue of stretching and reduction of residual distortions. In such cases, the gamma hyper parameter was added to the distortion parameter value estimated by the proposed system and normal undistortion with full view was performed. Both cases of undistorting the input using distortion parameters, with and without g hyper parameter are shown in Algorithm 2. Even in the worst-case distortion scenarios, the normal mode of undistortion yields the best output in default view as shown in the figure below. The normal undistortion mode outputs of the proposed method were significantly high in image quality and straight line constraint compared to the outputs from self-calibration methods such as [28,29].

**Algorithm 2:** Undistortion algorithm using distortion parameters, with and without γ hyper parameter

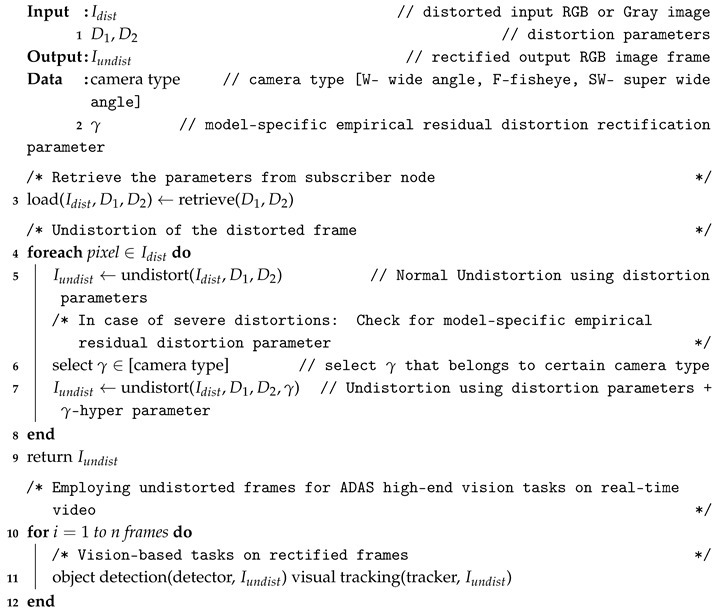



#### Possible Ways to Overcome Severe Distortions from Low-Quality Lens

This empirical study was deliberately carried out to overcome severe distortions from ultra-low-quality lens used on the ADAS workbench. In practice, researchers can adopt other possible ways to solve this issue as mentioned below rather than tuning parameters extensively through optical simulations and image-domain experiments.

- Employing a deep neural network to model the lens-specific distortions caused by the low-quality set of camera lens. Such an approach would be feasible for contextual usage in practical applications such as ADAS, video surveillance etc.
- An attempt of applying stretch-free super-resolution can be considered on the full view undistorted image to reduce the stretching along the image edges and to maintain pixel consistency.

In the future study, the model-specific empirical parameter approach can be replaced with deep-learning-based model-specific distortion rectification approach in conjunction with the proposed self-calibration method.

## 4. Experimental Data

The sensor systems with distinctive lens models such as fisheye, wide-angle, and super-wide-angle cameras were mounted on the ADAS platform for the real-time data acquisition and testing. Additionally, several tests have been conducted employing multiple cameras of all three main lens models i.e.; fisheye lens model cameras (5), wide-angle lens model cameras (6) and super-wide-angle lens model cameras (8). All the curated information regarding datasets, camera lens models, image sizes, distortion ranges, and data collection scenarios are briefly stated in Table 3 below.

### 4.1. Data Collection Scenarios

**Figure 12:**
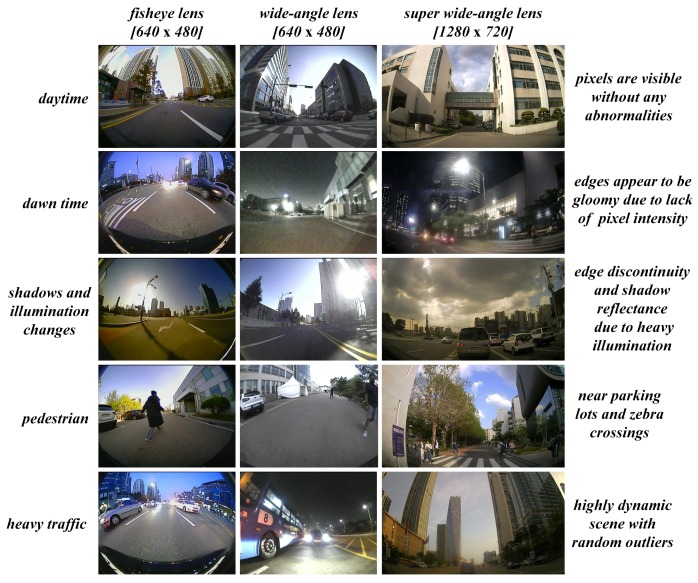
Data acquisition scenarios using various camera models.

The whole range of data samples was collected extensively using all the camera systems and precautions were taken such that the acquired data reflects an entire range of possible real-time scenarios as illustrated in Figure 12 below. Several aspects such as pedestrian, illumination changes, shadows, day time, dawn time, night time, heavy traffic, empty roads, parking lots, etc. were captured as a distinctive set of samples which were used to test the robustness of the proposed self-calibration method.

### 4.2. Synthetic Data Generation

**Figure 13:**
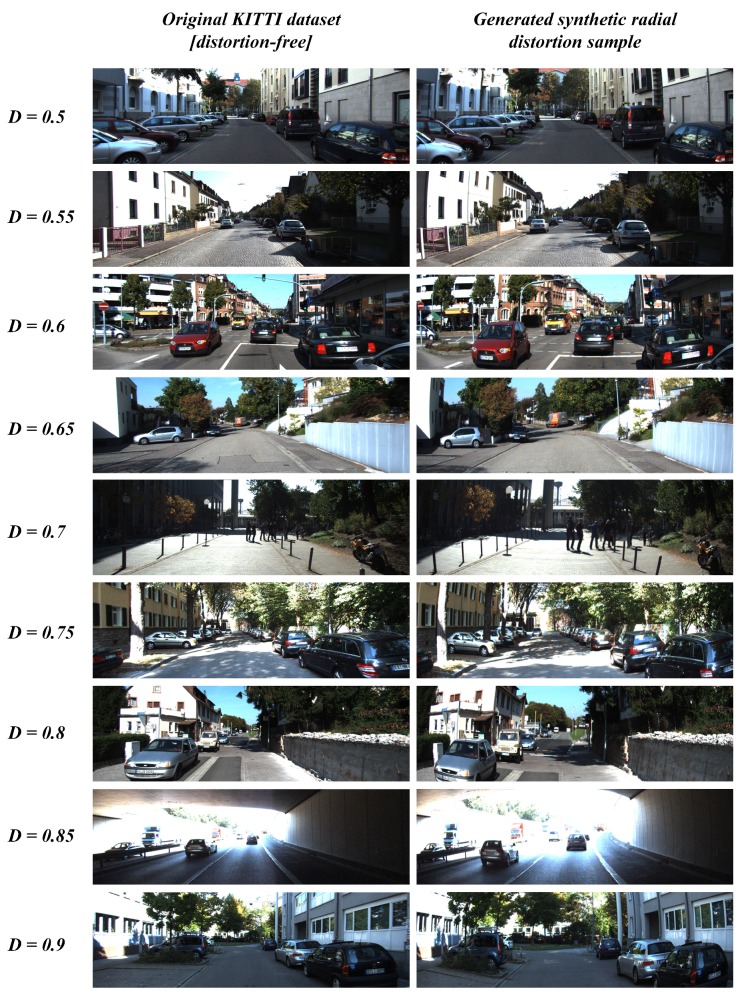
Synthetic distortion data generation using KITTI dataset.

The proposed system has been tested on the ADAS platform with multiple camera sensors bearing distinctive lens models. However, the robustness of the algorithm needs a more general testing scheme with evaluations on metrics such as PSNR and SSIM in terms of image quality. Accordingly, these metrics are universal modes of comparison tools in terms of pixel consistency which needs to be compared with an ultimate distortion-free sample. In other words, the original distortion-free sample is required to evaluate the self-calibration algorithm using PSNR and SSIM metrics by comparing the undistorted image with original distortion-free sample. Therefore, the synthetic data generation was carried out on a popular KITTI dataset and resulting samples were used for performance evaluation. The simulated images were presented in Figure 13 below and the motivation behind choosing KITTI dataset for synthetic data generation is because the use case context of this study is mostly focused on autonomous driving applications. Through this approach of synthetic data generation, the proposed method was tested on several random samples with distinctive range of distortion values. Additionally, the efficiency of the algorithm can be best validated by observing the silent point-based metrics such as principal point position(center of distortion) estimation error. Therefore, an open synthetic dataset [27] with various center of distortion ground truths were used for qualitative analysis of distortion center estimation.

## 5. Performance Analysis and Evaluation Metrics

The proposed method was extensively tested and validated using various evaluation metrics as well as the performance of the undistortion has be analyzed through multiple assessments. Firstly, the method was subjected to quantitative analysis using PSNR and SSIM metrics against both traditional pattern-based calibration and self-calibration methodologies on a distinctive range of synthetic generated data. Secondly, an additional quantitative analysis of principal point position estimation against popular self-calibration approaches has been made using open synthetic dataset with distortion center ground truths. Furthermore, the qualitative analysis has been carried out on the ADAS platform such that the algorithm’s output (undistorted frame) was tested by employing vision-based tasks such as object detection (reduction of miss-detection rates caused by lens distortions), vSLAM pipeline (point-cloud consistency), auto-parking (reprojection error). The performance analysis using these evaluation metrics covered a whole range of validations in terms of accuracy, feasibility with high-end pipeline tasks on ADAS platforms, etc.

### 5.1. Quantitative Analysis: Image Quality

The quantitative performance evaluation of the proposed system was performed against traditional OpenCV pattern-based calibration method and popular self-calibration methods such as Bukhari et al. [27] and Santana-Cedrés, Daniel et al.[28]. The metrics used to evaluate the output accuracy were PSNR and SSIM [43] on a pool of random distorted samples.

### 5.2. Quantitative Analysis: Salient Point Estimation

**Figure 14:**
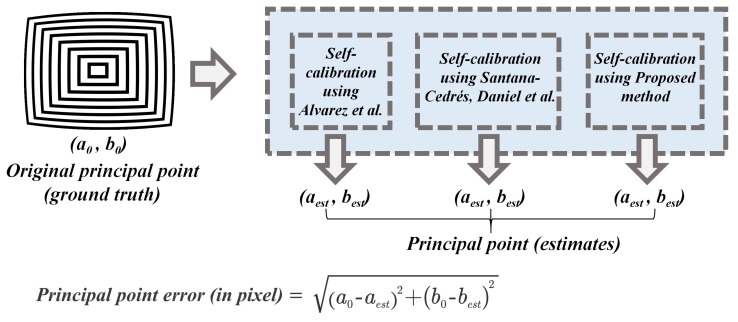
Determining Error in Silent point position (distortion center) estimation.

The salient point estimation such as calculation of principal point position (center of distortion) was performed using proposed method alongside most popular self-calibration techniques [28,29]. The synthetic image is fed into each self-calibration algorithm and the outputs of the algorithms were analyzed to note the distortion center position estimates. As the distortion center ground truth is available, the error in estimates was calculated using the Euclidean distance between the ground truth and estimated point position as shown in Figure 14 below.

### 5.3. Qualitative Analysis

The qualitative comparison can be considered to be a more pragmatic way of testing the proposed system directly on the ADAS platform alongside vision-based tasks such as object detection and localization etc. In this study, the output (undistorted frame) is subjected to high-end vision-based tests, such that the undistorted frame must improve the performance of those tasks in terms of efficiency which can be considered to be a qualitative metric. In the case of object detection, to maintain the consistency: the pre-trained model of YOLOv2 was considered to run on the undistortion frame as well as the distorted frame. As the original distortion image suffers from severe distortions and the undistorted output rectifies it to a significant level, the object detector is expected to perform well in detecting objects in the scene especially along the edges where the distortion is heavily rectified. Theoretically, such object detection results were depicted in terms of mAP (mean average precision) but that cannot be used in the case of qualitative comparisons between distortion and undistortion samples over object detection. Because the trained ground truth is different from the samples in this study, which might induce a bias towards a specific set of samples. Therefore, the qualitative comparison was carried out such that the random 100 samples from each camera model (fisheye, wide-angle, and super-wide-angle) were selected and undistorted. Therefore, a pre-trained object detector was deployed on distorted samples as well as undistorted samples. The qualitative metric used here was the “reduction of miss detections caused by lens distortions”. Precisely, the detection results (miss-detection cases) of distorted samples with deformations caused by lens distortions along the edges (top, bottom, and sides) were considered. Among which the percentage of instances that undistortion detection result actually improved the object detection and reduced the miss detections caused in distorted regions (edges: top, bottom, and sides) were recorded.

Similarly, the localization and mapping visual attributes were investigated on the output frames with a qualitative comparison by point-cloud consistency and reprojection error. The logical explanation over this choice is: the ADAS platform is often equipped with multiple sensors on-board among which the rear camera is one responsible for auto-parking and reverse collision control. It seems to have a potential application such as localization (retrieving vehicle’s pose in the environment) and mapping (building a map based on point-cloud). The localization and mapping tasks can be applied to almost all the camera sensors on-board such that point-cloud mapping assists the localization of the vehicle and thereby generating next point-cloud for mapping and so on. By including the experimentation of approaches such as visual structure from motion (vSfm) and visual SLAM, the point-cloud consistency can be visually compared in both scenarios (distortion and undistortion samples). In this study, the visual Sfm was employed to get the sequence of images in real time and render them to produce the 3D point-cloud for visual comparison. The visual SLAM framework used in the study was structured using traditional OpenCV pipeline functions such as Shi-Tomasi, Good Features to Track, Optical flow, PnP-RANSAC, and sparse bundle adjustment. In addition, the real-time testing includes using the fisheye rear-view camera for the auto-parking scenario, wide-angle front view camera for multiple-object tracking and super-wide-angle front view camera for localization and mapping. The metric considered here for the qualitative analysis was “average reprojection error (in pixels)” on the set of randomly selected 100 observations.

## 6. Results and Discussions

The quantitative and qualitative analysis has been carried out extensively using the above-described performance metrics, and qualitative testing on various data samples acquired from different real-time conditions. The results were acquired from tests conducted under diverse scenarios focusing on various aspects such as image size, distortion levels, environmental conditions, and performance on real-time vision tasks, etc.

### 6.1. Quantitative Analysis on the Synthetic Dataset

#### 6.1.1. PSNR and SSIM Metrics on the Synthetic Dataset

**Figure 15:**
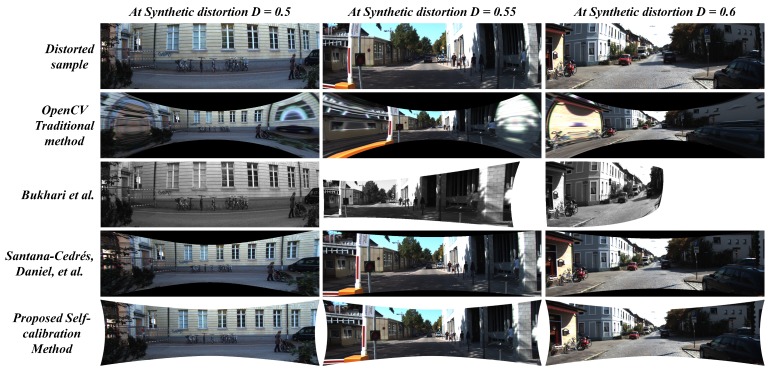
Comparison between proposed method against contemporary self-calibration and OpenCV traditional algorithms on Synthetically distorted KITTI data (D = 0.5∼0.6).

**Figure 16:**
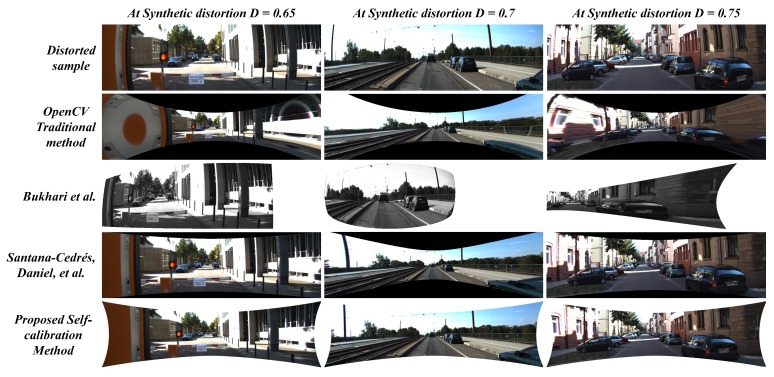
Comparison between proposed method against contemporary self-calibration and OpenCV traditional algorithms on Synthetically distorted KITTI data (D = 0.65∼0.75).

**Figure 17:**
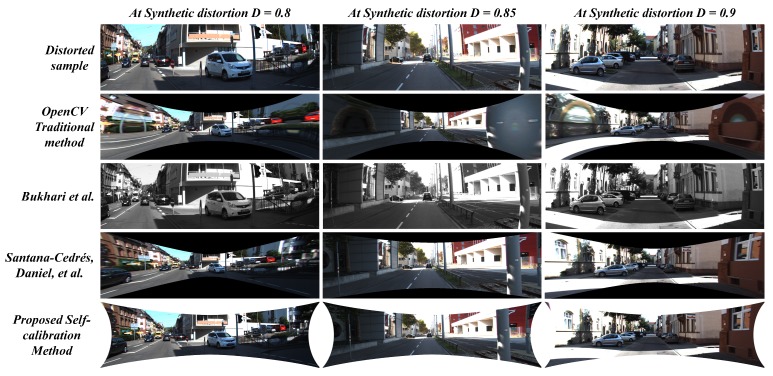
Comparison between proposed method against contemporary self-calibration and OpenCV traditional algorithms on Synthetically distorted KITTI data (D = 0.8∼0.9).

The Figure 15, Figure 16 and Figure 17 illustrates the comparative analysis of distortion rectification methodologies on synthetic data in correspondence to the diverse range of distortion levels. From the results, there are two major issues to be discussed: 1. The effect of undistortion on stretching to compensate the pixel distribution against the radial distortion and 2. Appearance correlation between the structural symmetry of the undistortion against the distortion-less original scene in terms of human perception.

The stretching factor has been significantly handled in the case of the proposed system and the two-parameter model proposed by Santana-Cedrés, Daniel et al. [28]. Although in a few cases, [28] method was not able to handle certain distortion levels due to its internal tuning of various hyper parameters in the algorithm. In the proposed system, the usage of reliable loss constraint and suitable undistortion scheme prevented the stretching effect along the edges of the image. The proposed system performed significantly well in preserving the edge pixels while compensating the radial distortion, whereas the OpenCV method and Bukhari et al. [27] method struggle to overcome this issue. On the other hand, the image quality metrics such as PSNR and SSIM were examined to estimate the appearance correlation. The proposed method attained high signal-to-noise ratio and structural similarity index in 8 out of 9 distortion levels tested on KITTI dataset. In a few cases, with best-tuned parameters in every intermediate step, the Santana-Cedrés, Daniel et al. [28] method attained high metric at one distortion level. The corresponding values of PSNR and SSIM metrics can be observed in Table 4.

#### 6.1.2. Principal Point Estimation on the Synthetic Dataset

The principal point estimation was carried out by various self-calibration algorithms alongside proposed method and the results were calculated and depicted in Table 5.

The average pixel error in the case of Alvarez et al. [29] and Santana-Cedrés, Daniel et al. [28] is high compared to the proposed method of self-calibration.

### 6.2. Practical Testing on ADAS Workbench Alongside Vision-Based Tasks

**Figure 18:**
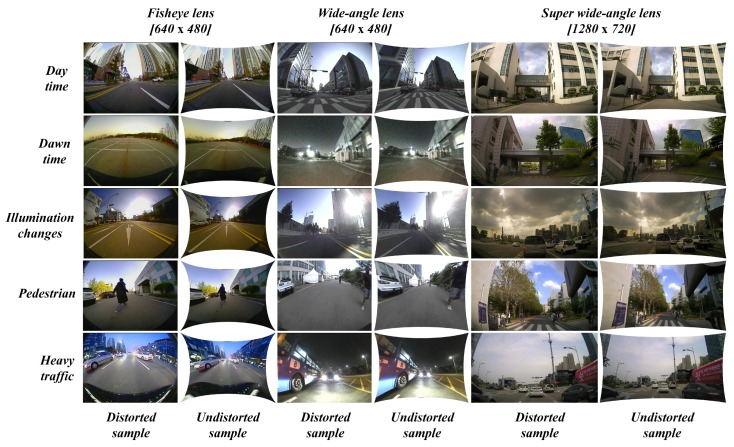
Performance of proposed algorithm on different camera acquisitions in various real-time scenarios.

The qualitative performance visualization of the extensive testing on various different portfolios are shown in Figure 18.

Various conditions were explored such as daytime, dawn time, illumination changes with shadows, reflections, and clouds as well as diverse driving conditions such as pedestrian, empty roads, heavy traffic, and inside the campus, etc. The figure consists of samples from variety of camera lens models with different distortion ranges. Almost all the samples were undistorted using the default mode of undistortion. Only two samples in the fisheye lens under daytime and heavy traffic were undistorted using hyper parameter along with the distortion parameters. This optional choice was made to reduce the stretching factor along the edges of the image due to severe lens distortions. In the process of performing experiments, few significant observations were made such as the proposed system was able to perform in environments with fewer straight lines as well as in dawn and night conditions with complex objects in the scene. On the other hand, the proposed system has limitations of handling ultra-low-cost lens images at lower resolutions with severe distortions. Although the proposed method performs well in undistorting severe distortions in default view, it suffers displaying all the pixels due to stretching anomaly along the edges in full view.

**Figure 19:**
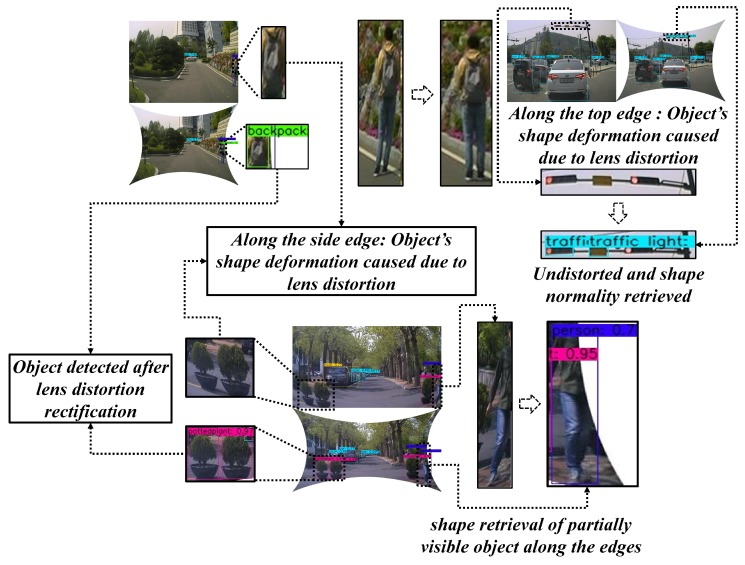
Real-time testing of lens-distortion rectification algorithm on object detection task.

For the better performance validation of the proposed system, a pragmatic approach of qualitative analysis has been conducted by testing the algorithms on the camera sensors mounted on the ADAS workbench. All three types of sensors were interchangeably positioned on different viewpoints on the vehicle to attain the best coverage of the scene for tasks such as object detection, localization, and mapping, auto-parking, etc. The results documented below consists of real-time vision tasks such as object detection on all the three kinds of cameras, multiple-object tracking on wide-angle camera, auto-parking on fisheye camera, localization, and mapping on super-wide-angle cameras. In the case of Figure 19, the object detection task had a severe disruption from the lens distortions causing object shape deformation.

The clear analogy can be shown between the lens distorted sample and miss-detection of a few objects due to those distortions. In the figure, the clear illustration of the objects such as backpack (side edge), traffic light (top edge), person (side edge) and flowerpots (bottom edge) being heavily susceptible to the lens-distortion deformations can be observed. Therefore, a random sample of 100 observations was taken into consideration and the percentage of miss-detection instances due to lens distortions and their successful detections on undistortion frames were recorded along the edges (top, bottom, and sides) on all three sensor types (fisheye, wide-angle and super-wide-angle). The results depicted in Table 6 clearly states the relativity between the object deformations caused due to lens distortions and miss-detection instances. For example, in the random collection of 100 fisheye samples: there were 41 cases of miss-detection due to disruption in shape along the right side of the frame which were successfully detected after the undistortion. This was applied for all the four edges on the data samples and percentage of coinciding well detection instances on undistortion frame with respect to miss-detection instances on the distorted frames were shown.

**Figure 20:**
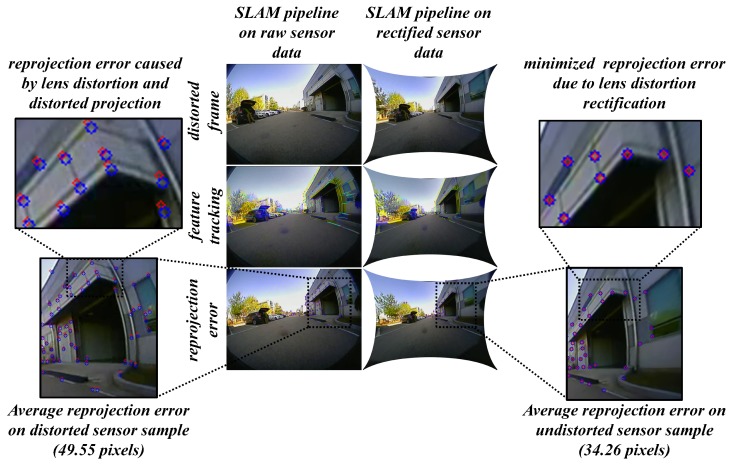
Auto-parking scenario on rear fisheye camera: Real-time visual SLAM pipeline on lens distortion rectified sensor data.

The localization and auto-parking tasks highly depend on the low-level features and their correspondences which means the scene must be clear without distortions such that the feature search can be reliable, and errors can be reduced. In case of lens distortions, the projections tend to bend and the features from the previous frames cannot correspond to the same feature points in the next frame, causing the reprojection error. In Figure 20, a clear illustration of this phenomenon can be observed in a fisheye frame with high average reprojection error in the distorted case than that of the undistorted case.

The fisheye frames of 100 observations were considered on a visual SLAM pipeline and the average reprojection error was recorded. The same approach has been followed using the other camera sensor models mounted in various positions on the vehicle deploying tasks such as multiple-object tracking, localization, and mapping. The average reprojection error in pixels was recorded and is illustrated in Table 7 For instance, the 100 frames of fisheye camera sensor mounted on the rear position of the vehicle exhibits 49.55 pixels of average reprojection error while performing the auto-parking task and the undistortion counterpart exhibits 34.26 pixels of average reprojection error.

**Figure 21:**
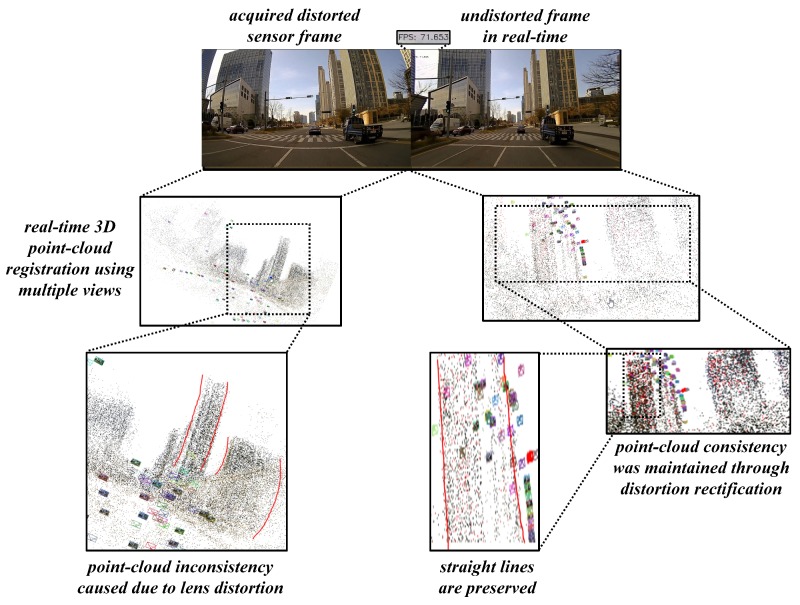
Real-time 3D point-cloud consistency testing on super-wide-angle undistorted data.

The reprojection error causes a chain of disruptions in the pipelines such as visual structure from motion (vSfM) and vSLAM in terms of localization errors and 3D mapping inconsistencies. The super-wide-angle camera sensor was employed and mounted on the front position of the ADAS workbench such that it captured the feature correspondences while the vehicle is moving and generates a 3D point-cloud based on feature correspondences. The results shown in Figure 21 proves that point-cloud consistency in undistortion sample was well-preserved when compared to that of the distortion sample. Especially the straight lines along the edges of the building can be observed to be deformed in the case of distortion sample and were successfully able to retrieve in the case of undistortion.

## 7. Conclusions

This paper proposes a self-calibration design to rectify the distortions over multiple larger FOV cameras used in the ADAS system. The proposed method with cross-entropy loss on novel straightness constraint, distortion parameter estimation, and undistortion module exhibit robustness in various conditions such as daytime, dawn time, illumination changes with shadows, reflections, clouds, pedestrians, heavy traffic, etc. The proposed method was evaluated through various experiments and metrics on both real-time data from ADAS system and synthetically generated distortion data samples. The proposed method performed well at various quantitative comparisons with traditional and contemporary state-of-the-art self-calibration methods on image quality and salient point position metrics. Additional aspects such as parameter sharing and model-specific empirical γ-residual rectification factors were invested experimentally. On the other hand, the proposed system has limitations of handling ultra-low-cost lens images with lower resolutions and severe distortions. Although the proposed method performs well in undistorting severe distortions in default view, it suffers displaying all the pixels due to stretching anomaly along the edges in full view. Overall system output was extensively tested by employing high-end vision-based tasks such as object detection, localization, mapping, and auto-parking on the undistorted frames in real time. The usage of distortion rectification algorithm on various kinds of lens models employed on different high-end vision tasks has significantly increased the reliability of the ADAS system compared to the case of distorted raw sensor samples. Also, this distortion rectification study can be further continued in conjunction with deep-learning and high-end vision tasks to explore new frontiers in complex concepts such as stretch-free undistortion, distortion-free depth extraction, and smallest object detection over a large scale regions using larger FOV cameras, etc.

## Figures and Tables

**Table 1:** Ablation study validating the attributes in proposed system.

Approach (Optimization Technique + Loss)	Performance		Parameter Estimation Processing Time (in Sec.)
	Average SSIM	Average Error in Principal Point Estimation (in Pixels)	
NR-optim + M-S loss	0.27	32.6	4.51
NR-optim + L-S loss	0.20	40.7	4.47
NR-optim + C-E loss	0.28	30.9	4.42
LM- optim + M-S loss	0.22	37.2	3.92
LM- optim + L-S loss	0.20	42.4	4.36
LM-optim + C-E loss	0.33	18.7	3.07

**Table 2:** Use case of parameter sharing scheme on high-resolution larger FOV image frames.

Image Size (in Pixels)	Field of View [FOV] (in Degrees)	Parameter Estimation Time (in Sec.)	
		Normal Mode	Parameter Sharing	
2880 × 1620	140	32.95	22.04	
5120 × 2880	200	64.92	54.47	
7680 × 4832	190	58.8	40.11	

**Table 3:** Summary of datasets and their attributes used in the experiments.

Dataset Type	Camera Lens Model	Image Size (w × h)	Distortion Ranges	Data Collection scenarios
Real data	Wide-angle Fisheye Super-wide-angle	1280 × 720 (HD) 640 × 480 (VGA) 320 × 240 (QVGA)	Distortions induced by lens models with FOV ranging from 120${}^{\circ }$ < FOV < 200${}^{\circ }$	Daytime, Dawn time, Illumination changes, pedestrians, heavy traffic
Synthetic data	Normal lens with FOV less than 100${}^{\circ }$	1242 x 375 default size of the KITTI data samples	Synthesized from D = 0.5 to 0.9 in the intervals of 0.05	On road with various objects such as cars, pedestrians, van, cyclist etc.

**Table 4:** PSNR and SSIM metrics for various methods on synthetic distortions on KITTI dataset.

Synthetic Distortion	OpenCV Traditional Method		Bukhari et al. [27]		Santana-Cedrés, Daniel et al. [28]		Proposed Method	
	PSNR	SSIM	PSNR	SSIM	PSNR	SSIM	PSNR	SSIM
D = 0.5	9.495	0.1347	16.259	0.217	20.826	0.403	21.16	0.425
D = 0.55	8.733	0.217	13.203	0.285	17.453	0.332	19.753	0.484
D = 0.6	8.591	0.229	14.103	0.201	21.738	0.498	20.062	0.471
D = 0.65	7.941	0.155	12.536	0.301	17.386	0.513	19.648	0.543
D = 0.7	8.023	0.321	14.414	0.421	16.453	0.568	21.05	0.612
D = 0.75	10.101	0.236	14.693	0.281	17.432	0.367	18.354	0.414
D = 0.8	9.21	0.206	12.28	0.321	16.15	0.405	16.343	0.411
D = 0.85	8.712	0.315	14.77	0.405	17.96	0.467	18.152	0.472
D = 0.9	7.957	0.249	10.295	0.299	12.83	0.335	16.694	0.421

**Table 5:** Estimation Error of principal point (center of distortion) using various self-calibration techniques on synthetic dataset.

Synthetic Image Principal Point (Original)	Alvarez et al. [29]		Santana-Cedrés, Daniel et al. [28]		Proposed Method	
	Principal Point (Estimate)	Error (in pixel)	Principal Point (Estimate)	Error (in Pixel)	Principal Point (Estimate)	Error (in Pixel)
(320,240)	(330,235)	0	(320,240)	0	(320,240)	0
(330,250)	(332,257)	21.3	(320,240)	14.1	(323,242)	10.6
(340,260)	(342,269)	32.2	(320,240)	28.3	(326,245)	20.5
(350,270)	(354,275)	28.4	(320,240)	42.4	(338,257)	17.6
(360,280)	(369,288)	17.0	(346,268)	18.4	(357,264)	16.2
(370,290)	(378,296)	47.4	(358,273)	20.8	(361,272)	20.1
(380,300)	(392,311)	34.4	(361,273)	33.0	(364,274)	31.5
(390,310)	(406,321)	49.5	(369,276)	39.9	(376,284)	29.8
**Average pixel error**	**28.8**		**24.5**		**18.7**	

**Table 6:** Percentage of miss-detection instances along the edges due to severe lens distortion which were rectified in the undistortion case.

Camera Model	Miss-Detection Percentage Along the Edges Due to Distortion (Number of Observations = 100)			
	Right Side	Left Side	Top	Bottom
Fisheye	41%	34%	37%	29%
Wide-angle	24%	19%	22%	26%
Super-wide-angle	36%	25%	27%	32%

**Table 7:** Average reprojection error: Real-time testing in the context of vision-based scenarios on Distortion and Undistortion data from various sensor models.

Camera Model (Position)	Vision-Based Scenario (Testing)	Average Reprojection Error (in Pixels) (Number of Observations = 100)	
		Distorted Data	Undistorted Data
**Fisheye (rear)**	Auto-parking	49.55	34.26
**Wide-angle (front)**	multiple-object tracking	48.17	30.64
**Super-wide-angle (front)**	localization and mapping	45.54	9.28

## Appendix A. Investigation of Distortion Distributions Corresponding to the Camera Models

During the experimental study, multiple camera models such 5 fisheye, 6 wide-angle and 8 super-wide-angle cameras with diverse FOV ranges were employed. The distortion ranges of those sensor units were estimated to investigate the practical difference between the three types of lenses. The distortion distribution of the cameras used are shown in Figure A1, where the upper part of the figure depicts the fluctuations in distortion parameters $D_{1}$ and $D_{2}$ with respect to the camera sensor. The bottom figures illustrated the distortion distribution and spread of $D_{1}$ and $D_{2}$ values corresponding to different models. During the testing, a few camera sensors exhibited diverse distortion values as shown in the upper part of the Figure A1. The distortion values marked with “red-square” correspond to the fisheye camera sensor which has different distortion value from the rest of the sensor unit bearing same fisheye model. Similarly, distortion values marked with “blue-triangle” and “black-circle” correspond to the wide-angle and super-wide-angle camera units with drastically deviated distortion values compared to the other camera units of same model respectively.

Although the three types of lenses are classified into separate models, they fall under a single larger FOV lens-distortion pattern of heavy distortions along the edges and comparatively low at the center. Apart from the real data distortion distributions, the proposed system was tested on various synthetic larger FOV distortion distributions to attain the best possible output compared to the current state-of-the-art self-calibration methods.
